# Cholesterol-raising diterpenes in types of coffee commonly consumed in Singapore, Indonesia and India and associations with blood lipids: A survey and cross sectional study

**DOI:** 10.1186/1475-2891-10-48

**Published:** 2011-05-15

**Authors:** Nasheen Naidoo, Cynthia Chen, Salome A Rebello, Karl Speer, E Shyong Tai, Jeanette Lee, Sandra Buchmann, Isabelle Koelling-Speer, Rob M van Dam

**Affiliations:** 1 Centre for Molecular Epidemiology, Yong Loo Lin School of Medicine, National University of Singapore, #05-02, 28 Medical Drive, 117456, Singapore; 2 Life Sciences Institute, Centre for Life Sciences, National University of Singapore, #05-02, 28 Medical Drive, 17456, Singapore; 3 Technische Universität Dresden, Department of Food Chemistry, Bergstr. 66, D-01062 Dresden, Germany; 4 Department of Medicine, National University of Singapore, 1E, Kent Ridge Road, NUHS Tower Block Level 10, 119228, Singapore; 5 Department of Epidemiology and Public Health, Yong Loo Lin School of Medicine, National University of Singapore; Block MD3 #03-17, 16 Medical Drive, 117597, Singapore; 6 Departments of Nutrition and Epidemiology, Harvard School of Public Health, Boston, Massachusetts, 02115, USA

## Abstract

**Background:**

To measure the content of cholesterol-raising diterpenes in coffee sold at the retailer level in Singapore, Indonesia and India and to determine the relationship of coffee consumption with lipid levels in a population-based study in Singapore.

**Methods:**

Survey and cross-sectional study in local coffee shops in Singapore, Indonesia and India to measure the diterpene content in coffee, and a population-based study in Singapore to examine the relationship of coffee consumption and blood lipid levels. Interviews and coffee samples (n = 27) were collected from coffee shops in Singapore, Indonesia and India. In addition, 3000 men and women who were Chinese, Malay, and Indian residents of Singapore participated in a cross-sectional study.

**Results and Discussion:**

The traditional 'sock' method of coffee preparation used in Singapore resulted in cafestol concentrations comparable to European paper drip filtered coffee (mean 0.09 ± SD 0.064 mg/cup). This amount would result in negligible predicted increases in serum cholesterol and triglyceride concentrations. Similarly low amounts of cafestol were found in Indian 'filter' coffee that used a metal mesh filter (0.05 ± 0.05 mg/cup). Coffee samples from Indonesia using the 'sock' method (0.85 ± 0.41 mg/cup) or a metal mesh filter (0.98 mg/cup) contained higher amounts of cafestol comparable to espresso coffee. Unfiltered coffee from Indonesia contained an amount of cafestol (4.43 mg/cup) similar to Scandinavian boiled, Turkish and French press coffee with substantial predicted increases in serum cholesterol (0.33 mmol/l) and triglycerides (0.20 mmol/l) concentrations for consumption of 5 cups per day. In the Singaporean population, higher coffee consumption was not substantially associated with serum lipid concentrations after adjustment for potential confounders [LDL-cholesterol: 3.07 (95% confidence interval 2.97-3.18) for <1 cup/week versus 3.12 (2.99-3.26) for ≥ 3 cups/day; p trend 0.12].

**Conclusions:**

Based on the low levels of diterpenes found in traditionally prepared coffee consumed in Singapore and India, coffee consumption in these countries does not appear to be a risk factor for elevation of serum cholesterol, whereas samples tested from Indonesia showed mixed results depending on the type of preparation method used.

## Background

The prevalence and burden of cardiovascular disease (CVD) in Asia is increasing [1-5]. A major contributor to this increase is hyperlipidemia, a major modifiable risk factor for CVD in both Western and Asian populations [6]. However, potential detrimental health effects of commonly consumed types of coffee including effects on other cardiovascular risk factors such as blood lipid levels [7] should be considered in recommendations on coffee consumption.

In Scandinavian populations consumption of boiled unfiltered coffee was associated with higher serum cholesterol concentrations [8]. The diterpenes cafestol and kahweol have been found to be present in substantial amounts in boiled unfiltered coffee [9-11] comprising 10-15% of the lipid fraction of roasted coffee beans [9]. Cafestol, and to a lesser extent kahweol, have been shown to increase total cholesterol, low-density lipoprotein cholesterol (LDL-C) and triglyceride concentrations, without substantial effects on high-density lipoprotein cholesterol (HDL-C) concentrations [10,12,13]. It has been shown that the different coffee preparation and brewing methods affect the concentration of cafestol and kahweol compounds present in the final brew [7-9,11,14].

Drip-filtered coffee, which is the main type of coffee consumed in the United States and Western Europe, has been shown to retain negligible amounts of cafestol and kahweol and to have minimal influence on blood lipid concentrations [9,15,16]. The type of coffee beans used (Arabica more than Robusta) and the particle size of the grounds (fine more than coarse) have been shown to affect the concentrations of cafestol and kahweol in brewed coffee [16-18]. In contrast, decaffeination and different levels of roasting have little effect on the diterpene concentrations in the coffee brew [11].

**Figure 1:**
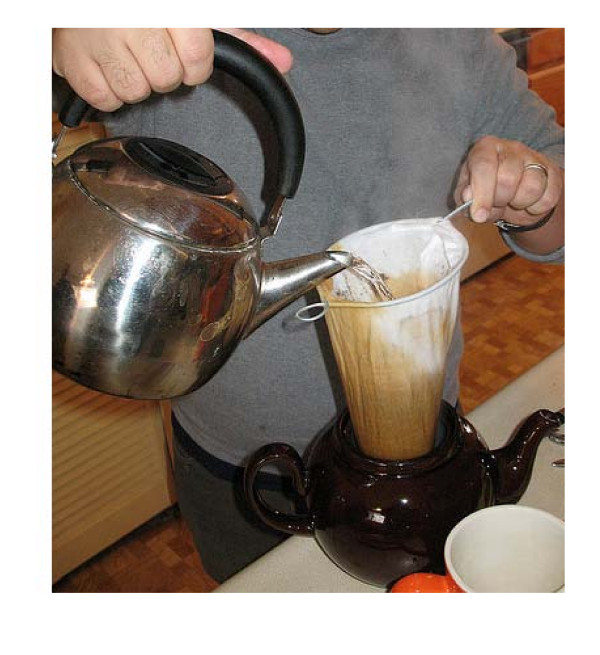
**Traditional 'Sock' Method used in Singapore and Malaysia**. (Used with kind permission of A. Cook, Ann Arbor, USA; http://www.flickr.com/photos/38668770@N00/372283340#/photos/amywcook/372283340/)

**Figure 2:**
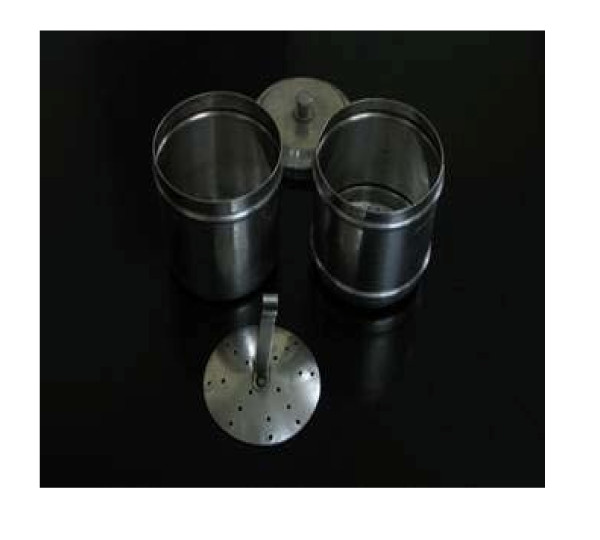
**South Indian metal 'filter' coffee instrument**. (Creative Commons Attribution-Share Alike 3.0 Unported license. http://en.wikipedia.org/wiki/Indian_filter_coffee)

Several Asian countries such as South India and Indonesia have long traditions of coffee cultivation, trade and consumption [19,20]. Coffee consumption in the Asian region has shown a steady increase over the past 20 years [21,22]. The diterpene levels in coffee consumed in Asian countries prepared using traditional methods such as the 'sock' method in Singapore and Malaysia (figure 1), the South Indian 'filter' coffee method (figure 2), and the coffee 'tubruk' method in Indonesia are unknown.

The traditional Singaporean and Malaysian 'sock' method involves the coffee beans being wok-roasted to a dark black brown with sugar, margarine and occasionally pineapple skin and corn and subsequently ground, brewed and filtered through the 'sock' into watering can-sized pots [23]. Nowadays, hot water is poured through the 'sock' into a receiving container and then pouring that fluid though the 'sock' again to constitute a single cycle. A number of cycles may be performed depending on taste. The South Indian 'filter' coffee method consists of an upper cup which has a pierced bottom, a pierced pressing disc with a central stem handle and a covering lid. This cup nests into the top of another round bottom cup, leaving enough room below to receive and contain the brewed coffee. The upper cup contains the coffee grounds mixed with chicory and the pressing disc compresses the grounds into a uniform layer across the pierced bottom of the upper cup. The upper cup is then placed into the top of the receiving cup and boiling water is poured inside. The lid is placed on top and the water is left to slowly drip into the receiving cup. The Indonesian 'tubruk' method consists of boiling coarse coffee grounds with solid sugar and serving it unfiltered, similar to the Turkish coffee method which uses finer grounds.

Because of the lack of data on the concentration of cholesterol-raising diterpenes in types of coffee commonly consumed in Asia, the relevance of coffee consumption for the prevention of cardiovascular disease in this region is unclear. Therefore, the aims of this current study were to 1) measure the diterpene content in coffee sold at the retailer level in Singapore, Indonesia and India and, 2) evaluate the association between coffee consumption and serum lipid levels in a multi-ethnic population-based study in Singapore.

## Methods

### Retailer Interviews and Coffee Sample Collection

We approached local coffee vendors and traditional '*kopitiams*' (coffee shops) in Singapore, Bangalore (South India) and Jakarta (Indonesia) for interviews and to obtain coffee samples. The *'kopitiam' *has a high prevalence in Singapore with several hundred present in and around the public housing estates, where approximately 85% of the population reside [23]. It was anticipated that the variability in coffee preparation methods would arise primarily from the type of establishment, namely *'kopitiams'*, compared to small restaurants which do not usually use traditional methods of preparation, and in Singapore, by ethnic location of the *'kopitiams'*. Efforts were made to interview vendors in the different ethnic locales in Singapore (Chinatown, Little India and Bugis/Geylang areas). In Jakarta and Bangalore, traditional coffee houses were approached ranging from street stalls to open cafes and small restaurants chosen by convenience sampling of vendors that were representative of common local establishments according to local guides. We included 14 coffee vendors (7 Chinese, 4 Malay and 3 Indian) in Singapore, 4 in Jakarta, and 5 in Bangalore.

Retail vendors were approached by 2 members of the research team, one of whom spoke the local language (Hokkien/Mandarin, Malay, Tamil, Kannada, or Bahasa) and the shop owners/supervisors were asked to participate in a short interview. Interviews were conducted in the first language of the participant. The background, aims and methods of the study were also explained to the participant prior to questionnaire administration. A standard cup of coffee (120 ml) was purchased for the sample collection.

The research instrument was an interviewer-administered standardised questionnaire assessing the following aspects of coffee preparation: (i) method (unfiltered, filtered) and types of filtration method used (paper, 'sock', metal), (ii) whether decaffeinated coffee was served, (iii) the use of additives to the coffee grounds (corn, margarine, chicory, none) and (iv) serving (milk+sugar, black). This study was approved by the National University of Singapore Institutional Review Board (NUS-IRB 10-066).

### Measurement of cafestol and kahweol in coffee

Coffee samples were collected from the selected coffee vendors and stored in polypropylene test tubes (4 × 50 ml tubes at approximately 50% capacity). Samples were subsequently deep frozen at -78°C until a sufficient number were present to freeze dry as a batch (5-7 days).

Samples were batch freeze dried in their original collection tubes for approximately 60 hours using a FreeZone 2.5 Plus, Model 7670030 (Labconco, Kansas City, MO, USA). The collector temperature of the system was -85°C and vacuumed at 0.016 mBar. The freeze dry chamber was covered in foil and cloth to block light as the diterpene compounds are photosensitive. After the first round of drying, all test tubes belonging to the same sample were combined into a single tube using an aliquot of 15 ml ethanol as a rinsing agent as diterpenes are located on the surface of the coffee brew and are lipid soluble. Hot water (15 ml) was applied to remove more resistant residue at the bottom of the tube. The combined sample was deep-frozen overnight at -78°C before the second round of freeze drying the following day.

**Figure 3:**
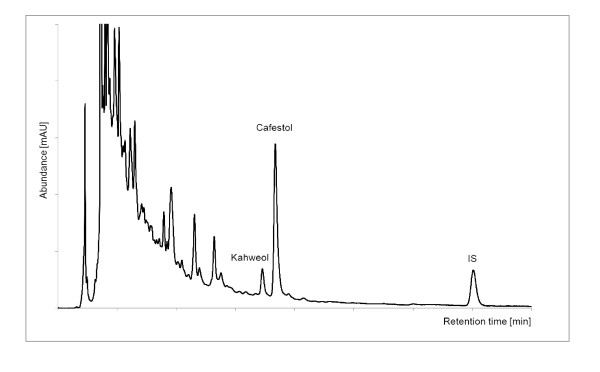
**HPLC chromatogram of a Singapore Chinese sample (λ = 224 nm)**.

The freeze dried samples were shipped under normal environmental temperature conditions and subject to temperature fluctuations which would not influence the subsequent analysis. The samples were analysed at the Institute of Food Chemistry, Technische Universität Dresden, Germany, using the modified DIN (German National Standard) 10779 method developed by Speer *et al *[24] for the quantitation of 16-O-methylcafestol in instant coffees. The coffee oil was saponified directly and the diterpene alcohols were determined in the unsaponified matter by reversed-phase high performance liquid chromatography (HPLC). This approved method was adapted to the freeze-dried coffee brews and validated for cafestol [17,18]. The kahweol content was calculated as cafestol applying the factor of 1,148 [25]. An internal standard was used to control the analyses. A typical HPLC chromatogram of a coffee brew is shown in Figure 3.

Predicted responses of coffee consumption on serum cholesterol and triglycerides were based on a meta-analysis conducted by Urgert and Katan (1997) of human trials with diterpene supplements which estimated that serum cholesterol increased by 0.13 mmol/l (5.0 mg/dl) for each 10 mg of cafestol and by 0.02 mmol/l (0.9 mg/dl) for each 10 mg of kahweol consumed per day [8]. Approximately 80% of this increase in serum cholesterol was due to increased levels of LDL-C, whereas no substantial effects were found on HDL-C. Triglycerides were also shown to be elevated by 0.08 mmol/l (7.3 mg/dl) for each 10 mg of cafestol and by 0.01 mmol/l (1.2 mg/dl) for each 10 mg of kahweol consumed per day for a period ranging from 2-6 weeks (11 trials) [8].

### Cross-sectional data from the Singapore Prospective Study 2 (SP2)

The SP2 study is a follow-up study of participants from 4 previous population-based studies in Singapore: the Thyroid and Heart Study from 1982-1984 [26], the National Health Surveys in 1992 [27] and 1998 [28] and the National University of Singapore Heart Study [29] over the period 1982 to 1998 [28]. The methods of these studies have been explained in detail previously [26-29]. A stratified random sampling method that oversampled ethnic minorities was utilised to increase representation of the Singaporean Malay and Indian ethnic groups. Fasting blood samples were collected from 5163 participants at clinic visits. For the present analysis, participants with pre-existing dyslipidemia (n = 1139), diabetes (n = 494), cardiovascular disease (n = 212), cancer (n = 56), reported race as 'other' (n = 2), were pregnant (n = 2), had implausible reported energy intakes (>7000 or <400 kcal) (n = 220), had missing covariate data (n = 19) and changed beverage intake in the past month preceding the interview (n = 35) were also excluded. Participants diagnosed with dyslipidemia were excluded as knowledge of the diagnosis and the disease, as well as the use of related medication may have affected their dietary choices and hence, this may reduce the ability to observe effects of other determinants. The final dataset size for analysis consisted of 3000 participants. This study was approved by the Singapore General Hospital Institutional Review Board (CIRB 2001/002/C). Written informed consent was obtained from all participants.

Data on coffee intake was obtained by asking participants about their habitual amount of coffee consumed. Participants could select one of 7 responses ranging from 'never/rarely' to '10 or more cups per day'. Coffee consumption was grouped into four categories (never/rarely, <1 cup/day, 1-2 cups/day and ≥ 3 cups per day) for the data analysis to avoid categories with small numbers.

Height was measured using a wall mounted measuring tape and weight was measured using a digital scale. BMI was calculated as weight (kg) divided by height squared (m^2^). Education level, physical activity and cigarette smoking were assessed using standardized questionnaires, whereas alcohol and dietary intakes were estimated based on responses to a validated Food Frequency Questionnaire [30]. Participants who used medications to lower cholesterol or triglycerides or increase HDL-C concentrations, or who reported having been diagnosed with hypercholesterolemia were considered to have a history of dyslipidemia. Participants who used anti-hypertensive medication or reported having been diagnosed by a physician with hypertension were considered to have a history of hypertension.

Total serum cholesterol, triglycerides and HDL-C concentrations were measured from fasting venous samples using enzymatic methods implemented in the Advia 2400 Chemistry System (Siemens Medical Solutions Diagnostics, Deerfield, IL, USA). Total serum cholesterol, triglycerides and HDL-C were measured using direct assays and LDL-C levels were calculated using the Friedewald formula [31].

The within-batch variations of coefficients (CV%) were 0.80%-1.57% for total serum cholesterol, 0.93%-1.15% for triglycerides and 0-3.85% for HDL-C. The between-batch CV% was 1.27%-3.40% for total serum cholesterol, 0.56%-0.65% for triglycerides and 1.18%-2.00% for HDL-C.

### Statistical Analysis

Statistical analysis of the SP2 study results was performed using the statistical packages STATA v10 (StataCorp, 2009) and SPSS v18 (SPSS, Inc., Chicago, Illinois). The dependent variables analysed were HDL-C, LDL-C, triglycerides and total serum cholesterol concentration. Natural logarithm transformations were performed for HDL-C and triglycerides, because the distribution for these variables was skewed. Geometric means were then obtained by taking the exponential of the means and the respective confidence intervals. Dependent variables outside 4 standard deviations were considered as outliers and excluded from the analysis.

Participant characteristics were compared across coffee consumption categories using the Kruskal-Wallis (for non-normally distributed continuous variables), analysis of covariance (for normally distributed continuous variables) or the chi-squared tests (for categorical variables). The potential confounders that we included in the multivariate models were selected *a priori *based on biological knowledge on determinants of serum lipid levels. These potential confounders were entered into the models one at a time to evaluate which had a substantial confounding effect on the association between coffee consumption and serum lipid concentrations leading to loss of statistical significance of associations. The first model included age (year), gender (male, female), and ethnicity (Chinese, Malay, Indian). The second model also included body mass index (BMI) (kg/m^2^), smoking (never smoker, past smoker, current <10 cigarettes/day, current ≥10cigarettes/day), education (primary and below, secondary, polytechnic and diploma, university), physical activity (kcal/week), hypertension (yes, no), alcohol intake (non-drinker, <1 serving/day, ≥1 serving/day). The third model included the above and energy intake (kcal), fiber (g/1000 kcal), cholesterol intake (mg/1000 kcal), polyunsaturated fatty acids (PUFA) intake (% of total energy), monounsaturated fatty acids (MUFA) intake (% of total energy) and saturated fatty acids (SFA) intake (% of total energy). Tests for trends were calculated modelling the medians of the coffee consumption categories as a continuous variable. All tests were 2-sided and P values < 0.05 were considered statistically significant.

## Results

### Preparation methods and diterpene concentrations

We evaluated the coffee preparation methods in 27 coffee retailers in Singapore, Indonesia and India where the coffee samples were collected (Table 1). All retailers used ground coffee as the main source with the exception of two Singapore-Indian retailers who used instant coffee. The filtration methods used varied between countries. All ground coffee in Singapore was filtered using the 'sock' (nylon/cotton mesh) method, whereas a metal mesh filter was used in India and a combination of the 'sock', metal mesh and unfiltered methods in Indonesia.

**Table 1:** Interview responses to coffee preparation methods from coffee retailers in Singapore, Indonesia and India (n = 27). Based on the requests of a standard cup of coffee (120 ml).

n (%)	Singapore-Chinese (n = 7)	Singapore-Malay (n = 5)	Singapore-Indian (n = 5)	Indonesia (Jakarta) (n = 4)	India (Bangalore) (n = 6)
Preparation Method					

Unfiltered	0	0	0	1	0

Filtered (metal mesh)	0	0	0	1	6

Filtered (sock)	7	5	3	2	0

Instant	0	0	2	0	0

De-caffeinated coffee	0	0	0	0	0

Additives					

Margarine	5	2	0	0	0

Corn	1	0	0	0	0

Chicory	0	0	2	0	5

None	1	3	3	4	1

Servings					

Milk + sugar	7	3	5	2	6

Black	0	2	0	2	0

Additives such as margarine and corn were added to the ground coffee at the distributor level and served at Singapore-Chinese and Singapore-Malay retailers only. Chicory was another additive in ground coffee served in Singapore-Indian and Indian retailers (70% coffee beans and 30% chicory in one sample). Samples from Indonesia did not contain additives (Table 1).

The diterpene concentrations of cafestol and kahweol in the collected coffee samples are shown in Table 2. In the Singaporean coffee samples prepared using the 'sock' filtration method, the overall mean concentrations of cafestol and kahweol were 0.09 mg and 0.03 mg per cup (120 ml) respectively. Similarly, in the coffee samples from South India using the metal mesh filtration method, the mean concentration of cafestol was 0.05 mg per 120 ml cup. These concentrations are similar to those found in paper drip filtered coffee and would lead to negligible increases in serum cholesterol and triglycerides for the consumption of 5 cups of coffee per day based on the prediction formulae by Urgert and Katan (1997) [8].

**Table 2:** Cafestol and kahweol concentrations of coffee samples and predicted effects on serum cholesterol for consuming 5 cups daily. Coffee samples are from Singapore, Indonesia, India (current study) and Europe (Urgert et al, 1995) using various brewing methods.

				**Predicted effect of 5 cups/day**	
**Source of Coffee Sample**	**Brewing Methods**	**Cafestol (mg/cup)**	**Kahweol (mg/cup)**	^#^**Serum cholesterol (mmol/l)**	^#^**Fasting triglycerides (mmol/l)**
**Current study (Asia)**					
**Singapore overall **(n = 14)	filtered ('sock')	0.09 ± 0.064 (0.02 - 0.23)	0.01 ± 0.02 (0.01 - 0.06)	0.01	<0.01
Singapore Chinese (n = 7)	filtered ('sock')	0.13 ± 0.07 (0.04 - 0.23)	0.02 ± 0.02 (0.00 - 0.06)	0.01	0.01
Singapore Malay (n = 4)	filtered ('sock')	0.06 ± 0.05 (0.02 - 0.13)	0.005 ± 0.01 (0.00 - 0.02)	0.01	<0.01
Singapore Indian (n = 3)	filtered ('sock') only	0.04 ± 0.02 (0.03 - 0.07)	0.003 ± 0.005 (0.00 - 0.01)	<0.01	<0.01
**India **(n = 5)	filtered (metal)	0.05 ± 0.04 (0.01 - 0.12)	0.03 ± 0.03 (0.01 - 0.07)	<0.01	<0.01
**Indonesia (overall) **(n = 4 )	filtered ('sock', metal); unfiltered	1.78 ± 1.78 (0.56 - 4.43)	0.24 ± 0.24 (0.09 - 0.59)	0.13	0.08
Indonesia (n = 2)	filtered ('sock')	0.85 ± 0.42 (0.56 - 1.15)	0.15 ± 0.08 (0.09 - 0.20)	0.06	0.04
Indonesia (n = 1)	filtered (metal)	0.98	0.09	0.07	0.04
Indonesia (n = 1)	unfiltered	4.43	0.59	0.33	0.20
**Urgert et al (1995) (Europe)**					
Scandinavian boiled (n = 14)*	boiled	2.4 ± 2.24 (0.64-9.68)	3.12 ± 2.72 (0.8-11.68)	0.18	0.11
Turkish/Greek (n = 11)*		3.12 ± 2.56 (0.4-8.0)	3.12 ± 3.12 (0.08-8.56)	0.23	0.14
French Press (n = 5)*	French press	2.8 ± 0.96 (1.84-4.4)	3.52 ± 1.68 (2.08-6.4)	0.21	0.13
Espresso:					
Italy (n = 10)*	espresso	1.2 ± 0.8 (0.16-2.32)	1.44 ± 1.04 (0.16-3.12)	0.09	0.05
Other (n = 21)*	espresso	0.96 ± 0.72 (0.0-2.48)	1.12 ± 0.88 (0.0-3.12)	0.07	0.04
Instant*	instant	0.2 (0.0-0.6)	-	0.01	0.01
Drip filtered (n = 3)*	filtered (paper)	0.08 ± 0.0	-	0.01	<0.01
Drip filtered (n = 3)*	filtered (nylon)	0.64 ± 0.08	-	0.05	0.03

Data are means ± SD (range). 1 cup is 120 ml.

*Adapted from Urgert et al (1995b); for some of the samples no SD or range for cafestol or no data on kahweol was presented in this publication.^# ^Based on the calculation proposed by Urgert and Katan (1997): Δcholesterol (mmol/l) = 0.015 × cafestol (mg/day) and Δtrigycerides (mmol/l) = 0.009 × cafestol (mg/day)

In the Indonesian coffee samples, using the 'sock' and metal mesh filtration methods, the mean concentrations of cafestol were 0.85 mg and 0.98 mg per 120 ml cup respectively. These concentrations are similar to the European espresso coffee samples but substantially higher than the Singapore 'sock' filtered samples. The cafestol concentration from the unfiltered coffee sample was 4.43 mg per cup which is equivalent to a predicted increase in serum cholesterol and serum triglycerides of 0.33 mmol/l and 0.20 mmol/l respectively for the consumption of 5 cups per day. These diterpene concentrations are similar to the high concentrations found in Scandinavian boiled, Turkish/Greek, and French press coffee samples.

### Coffee consumption and serum lipid concentrations in a Singaporean population

The majority of participants of the population-based study in Singapore consumed 1 to 2 cups of coffee per day (50.3%) and a lower proportion consumed 3 or more cups per day (8.1%). Table 3 shows selected demographic and dietary consumption characteristics of the participants according to coffee consumption. Coffee consumption was similar in the Chinese, Malay, and Indian ethnic groups. Higher coffee consumption was significantly associated with being male, older age, higher BMI, alcohol consumption, lower education level, cigarette smoking, higher saturated fat intake, lower polyunsaturated fat intake, and lower fibre intake (Table 3).

**Table 3:** Characteristics of the Singapore Prospective Study (SP2) (N = 3000) by coffee consumption categories.

Characteristic	Total	Never or <1 cup/week	<1cup/week to <1 cup/day	1-2 cups/day	≥3 cups/day	P value
		**n = 891**	**n = 358**	**n = 1509**	**n = 242**	

Age (y)	47.5 ± 11.2	45.3 ± 11.5	45.9 ± 11.4	49.0 ± 10.9	48.5 ± 10.1	<0.001

Gender						

Male	1348 (44.9)	327 (36.7)	159 (44.4)	689 (45.7)	173 (71.5)	
	
Female	1652 (55.1)	564 (63.3)	199 (55.6)	820 (54.3)	69 (28.5)	<0.001

Ethnicity						

Chinese	2058 (68.6)	575 (64.5)	254 (70.9)	1063 (70.4)	166 (68.6)	
	
Malay	531 (17.7)	183 (20.5)	62 (17.3)	243 (16.1)	43 (17.8)	
	
Indian	411 (13.7)	133 (14.9)	42 (11.7)	203 (13.5)	33 (13.6)	0.074

BMI (kg/m^2^)	23.6 ± 4.3	23.4 ± 4.3	23.4 ± 4.6	23.7 ± 4.4	23.9 ± 3.9	0.159

Education						

Primary school and below	705 (23.5)	163 (18.3)	75 (20.9)	395 (26.2)	72 (29.8)	
	
Secondary school	1295 (43.2)	378 (42.4)	131 (36.6)	681 (45.1)	105 (43.4)	
	
Polytechnic and diploma	518 (17.3)	189 (21.2)	67 (18.7)	225 (14.9)	37 (15.3)	
	
University	482 (16.1)	161 (18.1)	85 (23.7)	208 (13.8)	28 (11.6)	<0.001

Physical Activity (kcal/week)	523.1 (268.9-952.7)	522.3 (251.5-987.2)	481.0 (268.0-826.4)	530.8 (277.0-937.2)	550.5 (273.3-1108.2)	0.141

Energy intake (kcal)	1829.8 (1443.9-2336.6)	1788.1 (1386.2-2278.7)	1763.8 (1387.4-2324.7)	1824.6 (1465.2-2304.1)	2132.6 (1666.6-2647.2)	<0.001

Fiber intake (g/1000 kcal)	10.5 (9.1-12.3)	10.9 (9.3-12.8)	10.6 (9.3-12.4)	10.5 (9.1-12.2)	9.1 (7.9-10.8)	<0.001

Cholesterol intake (mg/1000 kcal)	115.4 (90.2-141.7)	115.2 (88.8-142.7)	110.9 (88.1-140.6)	116.2 (91.0-140.3)	119.6 (93.0-149.3)	0.171

PUFA intake (% of energy)	5.3 (4.2-7.2)	5.5 (4.2-7.6)	5.3 (4.2-7.1)	5.3 (4.2-7.2)	4.8 (3.9-6.4)	0.001

MUFA intake (% of energy)	9.7 ± 2.6	9.8 ± 2.7	9.8 ± 2.5	9.6 ± 2.6	9.5 ± 2.5	0.001

SFA intake (% of energy)	11.1 ± 2.8	11.1 ± 3.0	10.9 ± 2.6	11.1 ± 2.8	11.6 ± 2.8	<0.001

Cigarette Smoking						

Never smoker	2393 (79.8)	782 (87.8)	298 (83.2)	1184 (78.5)	129 (53.3)	
	
Past smoker	240 (8.0)	47 (5.3)	24 (6.7)	139 (9.2)	30 (12.4)	
	
Current (<10 cigarettes/day)	115 (3.8)	26 (2.9)	15 (4.2)	62 (4.1)	12 (5.0)	
	
Current (≥10 cigarettes/day)	252 (8.4)	36 (4.0)	21 (5.9)	124 (8.2)	71 (29.3)	<0.001

Alcohol Consumption						

non-drinker	2457 (81.9)	755 (84.7)	289 (80.7)	1227 (81.3)	186 (76.9)	
	
<1 serving/day	479 (16.0)	128 (14.4)	62 (17.3)	241 (16)	48 (19.8)	
	
≥1 serving/day	64 (2.1)	8 (0.9)	7 (2.0)	41 (2.7)	8 (3.3)	0.012

Abbreviations: PUFA - polyunsaturated fatty acids, MUFA - monounsaturated fatty acids, SAF - saturated fatty acids

Values are mean ± SD for continuous variables and n (%) for categorical variables.

The Kruskal-Wallis test (for non-normally distributed continuous variables), analysis of covariance (for normally distributed continuous variables) or the chi-squared test (for categorical variables) was used to compare participant characteristics across coffee consumption categories.

We further evaluated the association between coffee consumption and serum lipid concentrations (Table 4). After adjusting for age, gender and ethnicity, higher coffee consumption was associated with higher LDL-C, triglycerides and total cholesterol concentrations. After additional adjustment for potential confounders (Model 2) none of these associations remained statistically significant. The variables that were responsible for the weakening of associations leading to non-significant P-values for trend were BMI for one outcome (triglycerides) and the combination of BMI, education and smoking for other outcomes (LDL-C and total cholesterol). Therefore, in the fully adjusted model, coffee consumption was not significantly associated with any of the serum lipids (Table 4).

**Table 4:** Mean serum lipid concentrations in the SP2 study (N = 3000) according to level of coffee consumption

		Concentration (geometric mean (95% CI))					
			
	Fasting lipids, mmol/L	Never or <1 cup/week	1 cup/week to <1 cup/day	1-2 cups/day	≥3 cups/day	Percentage difference between extreme groups	P for trend
		n = 891	n = 358	n = 1509	n = 242		

**Model 1**‡	HDL-cholesterol	1.32 (1.30 - 1.34)	1.33 (1.30 - 1.36)	1.33 (1.32 - 1.35)	1.27 (1.23 - 1.31)	-3.79%	0.057
	
	LDL-cholesterol	3.19 (3.13 - 3.24)	3.14 (3.06 - 3.22)	3.26 (3.22 - 3.30)	3.31 (3.21 - 3.40)	3.76%	0.004
	
	Triglycerides	1.14 (1.10 - 1.18)	1.17 (1.11 - 1.23)	1.16 (1.13 - 1.19)	1.25 (1.18 - 1.34)	9.65%	0.020
	
	Total cholesterol	5.14 (5.09 - 5.20)	5.11 (5.02 - 5.20)	5.24 (5.19 - 5.29)	5.27 (5.16 - 5.38)	2.53%	0.005

**Model 2**‡ ‡	HDL-cholesterol	1.37 (1.34-1.41)	1.39 (1.34-1.43)	1.40 (1.37-1.43)	1.35 (1.31-1.40)	-1.46%	0.809
	
	LDL-cholesterol	3.05 (2.95-3.15)	3.00 (2.88-3.11)	3.10 (3.01-3.19)	3.11 (2.98-3.23)	1.97%	0.098
	
	Triglycerides	1.23 (1.16-1.30)	1.24 (1.16-1.33)	1.21 (1.14-1.28)	1.26 (1.17-1.36)	2.44%	0.762
	
	Total cholesterol	5.13 (5.03-5.24)	5.09 (4.96-5.21)	5.19 (5.09-5.29)	5.18 (5.04-5.32)	0.97%	0.163

**Model 3**‡ ‡ ‡	HDL-cholesterol	1.38 (1.34-1.42)	1.39 (1.35-1.43)	1.40 (1.37-1.44)	1.36 (1.31-1.41)	-1.19%	0.976
	
	LDL-cholesterol	3.05 (2.96-3.15)	3.00 (2.88-3.11)	3.10 (3.01-3.19)	3.10 (2.98-3.23)	1.69%	0.120
	
	Triglycerides	1.22 (1.15-1.30)	1.24 (1.15-1.33)	1.20 (1.14-1.27)	1.24 (1.15-1.35)	1.52%	0.961
	
	Total cholesterol	5.14 (5.03-5.25)	5.09 (4.96-5.22)	5.20 (5.10-5.30)	5.18 (5.04-5.32)	0.78%	0.183

‡ Model 1: Adjustment for age (yr), gender (male, female), ethnicity (Chinese, Malay, Indian)

‡ ‡ Model 2: Adjustment for age (yr), gender (male, female), ethnicity (Chinese, Malay, Indian), BMI (kg/m^2^), smoking (non-smoker, ex-smoker, smoker <10cigarettes/day, smoker ≥10cigarettes/day), education (primary and below, secondary, polytechnic and diploma, university), physical activity (kcal/week), hypertension (yes, no), alcohol (non-drinker, <1serving/day, ≥ 1 serving day)

‡ ‡ ‡ Model 3: Adjustment for age (yr), gender (male, female), ethnicity (Chinese, Malay, Indian), BMI (kg/m^2^), smoking (non-smoker, ex-smoker, smoker <10cigarettes/day, smoker ≥10cigarettes/day), education (primary and below, secondary, polytechnic and diploma, university), physical activity (kcal/week), hypertension (yes, no), alcohol (non-drinker, <1serving/day, ≥ 1 serving day), energy intake (kcal), fiber (g/day/1000 kcal), cholesterol (mg/day/1000 kcal), PUFA (% of total energy), MUFA (% of total energy), SFA (% of total energy)

## Discussion

In our study, the traditional 'sock' method resulted in amounts of cafestol and kahweol comparable to European paper drip filtered coffee [11]. The Indian 'filter' coffee contained similarly low amounts of cafestol and kahweol. Both of these amounts would result in a negligible predicted increase in serum cholesterol and triglyceride concentrations (Table 2). Coffee samples from Indonesia using the 'sock' method and a metal mesh filter contained amounts comparable to espresso coffee [11]. Unfiltered coffee from the Indonesian sample contained amounts comparable to Scandinavian boiled, Turkish and French press coffee [11] which would result in substantial predicted increases in serum cholesterol. In the population-based study in Singapore, higher coffee consumption was not associated with elevated serum lipids after adjustment for potential confounders. This finding is consistent with the low cafestol levels found in coffee prepared in Singapore using the 'sock' method.

The higher concentration of cafestol and kahweol found in the Indonesian samples as compared with the Singapore samples using the 'sock' method could be explained by the different porosities of the material used to manufacture the sock, the number of times the brew was filtered through the sock and the density of the grounds used, i.e. how compacted the grounds were. The Indonesian metal mesh filter method resulted in substantially higher amounts of cafestol and kahweol than the Indian metal mesh 'filter' method. This higher concentration may be explained by the different porosities of the filters used and the number of times that the brew was filtered.

To our knowledge, the association between coffee consumption and serum lipid levels has not been previously examined in populations commonly using the 'sock' filter method. However, results from our cross-sectional study are consistent with previous cross sectional studies in Japanese and European populations that have shown no substantial association between filtered coffee that contains similar amounts of cafestol, and serum lipid levels [32-35]. In addition, a meta-analysis of 14 randomised controlled trials by Jee *et al *(2001) showed that filtered coffee resulted in minimal increases in serum cholesterol, whereas boiled or unfiltered coffee substantially increased serum cholesterol [36].

This study addresses the paucity of information on preparation methods and the resultant diterpene levels in several coffee types commonly consumed in Asian countries. For Singapore, we obtained consistent results for diterpene levels in the commonly used type of coffee and a lack of association between coffee consumption and serum lipid levels in a cross-sectional study. The cross-sectional design did not allow us to establish the temporality of events. However, exclusion of participants with known hyperlipidemia greatly reduced the likelihood that associations between coffee consumption and lipids levels were due to changes in coffee consumption after diagnosis of hyperlipidemia. Although the 'sock' method is a common method of coffee preparation in Singapore, another limitation of the population-based study is that the preparation method of the coffee consumed was not assessed. Also, as a result of measurement error or residual confounding our cross-sectional analysis cannot exclude small effects of coffee consumption of serum lipids. A further limitation of our study of coffee samples was the relatively small number of samples analysed. However, results for the Indian and Singaporean coffee samples were highly consistent and based on similar numbers of samples as a previous European study [11]. The results for Indonesia should be interpreted with more caution given the small number of samples per preparation method, but the concentration for boiled coffee was as expected based on data from other non-filtered types of coffee [11].

## Conclusions

Based on the low levels of diterpenes found in traditionally prepared coffee consumed in Singapore and South India, it appears that coffee consumption in these countries is not an important risk factor for the elevation of serum lipids. Samples tested from Indonesia showed mixed results depending on the type of preparation method used. In Indonesia and other countries where the consumption of unfiltered coffee is common, use of alternative coffee preparation methods may provide an opportunity for cardiovascular disease prevention.

## Competing interests

The authors declare that they have no competing interests.

## Authors' contributions

NN was involved in the study concept and design, acquisition, analysis and interpretation of sample data, drafting of the manuscript, provided administrative, technical and material support. CC was involved in study concept and design, acquisition, analysis and interpretation of sample and cohort data, drafting of the manuscript, statistical analysis and provided administrative, technical and material support. SAR was involved in the study concept and design and interpretation of data and critical revision of manuscript for intellectual content. KS, SB and IKS were involved in the acquisition, analysis and interpretation of biochemical data. EST and JL were involved in acquisition of population cohort data and critical revision of manuscript for intellectual content. RVD was involved in the study concept and design, acquisition, analysis and interpretation of sample and cohort data, critical revision of manuscript for intellectual content, obtaining funding and overall study supervision. All authors read and approved the final manuscript.
